# Exploration of physicochemical properties and molecular interactions between cellulose and high-amylose cornstarch during extrusion processing

**DOI:** 10.1016/j.crfs.2021.07.001

**Published:** 2021-07-08

**Authors:** Pichmony Ek, Bon-Jae Gu, Steven R. Saunders, Kerry Huber, Girish M. Ganjyal

**Affiliations:** a School of Food Science, Washington State University, Pullman, WA, 99164 -6376, USA; b The Gene and Linda Voiland School of Chemical Engineering and Bioengineering, Washington State University, Pullman, WA, 99164-6515, USA; c Faculty of Chemical and Food Engineering, Institute of Technology of Cambodia, Phnom Penh, Cambodia; d Department of Animal and Food Science, Brigham Young University-Idaho, Rexburg, ID, 83460, USA

**Keywords:** Starch, Cellulose, Extrusion cooking, Expansion, Molecular changes, Structural changes

## Abstract

Incorporating fiber at high levels (>10%) into direct-expanded products with acceptable texture is challenging. Fundamental explanations for the interaction of starch and fiber and the cause of expansion reduction need further understanding for the effective incorporation of fiber into expanded products. This study aims to explain how cellulose content impacts the physicochemical properties of starch-based extrudates and the long-range and short-range molecular changes of starch. Mixtures of cornstarch (50% amylose) and cellulose were extruded using a co-rotating twin-screw extruder. Thermal and pasting properties of the raw mixtures were evaluated, and the physicochemical properties and microstructure of extrudates were determined. Long-range and short-range molecular changes of starch-cellulose mixtures before and after extrusion were observed by X-ray Diffraction (XRD) and Fourier Transform Infrared (FTIR) spectroscopy. The expansion ratio of extrudates reduced significantly as the cellulose content increased and had a strong negative correlation with crystallinity. Cell structures of starch-cellulose extrudates had a smaller and more uniform pore size but possessing a more ruptured matrix. FTIR spectra suggested that there was no covalent bonding interaction between starch and fiber after extrusion. Extrusion reduced the overall crystallinity compared to the raw mixtures. XRD showed that the crystallinity of the starch-cellulose extrudates increased as the cellulose content increased, and the XRD peaks representing cellulose remained unchanged. Cellulose could interfere with starch chain reassociation through intermolecular hydrogen bonding during the expansion process. Phase separation of starch and cellulose is likely to occur at high cellulose content, which could be another reason for the reduced expansion.

## Highlights

• FTIR showed no covalent bonding interactions between starch and cellulose.
• Crystallinity of extrudates increased with increased cellulose content.
• Expansion is negatively correlated with crystallinity.
• Cellulose interferes with the reassociation of starch biopolymers.
• Phase separation of starch and cellulose is likely to occur during extrusion.

## Graphical abstract

**Figure:**
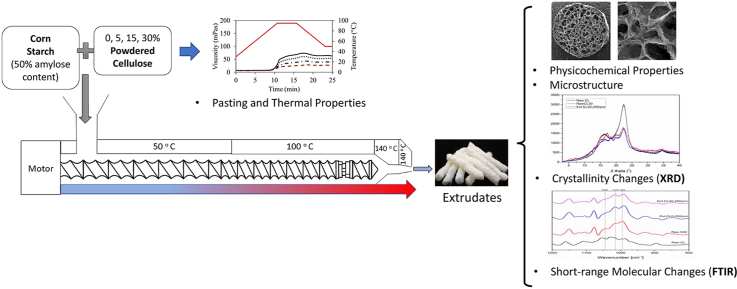

## 1 Introduction

Snacks and breakfast cereals are commonly produced by extrusion processing using the phenomenon of direct expansion. However, the production of high-fiber extruded products is still challenging, especially with those incorporating insoluble fiber. Many high-fiber foods are not well-accepted by consumers, mainly because of their poor sensory attributes (Robin et al., 2012). Refined flours or starches are commonly used to produce these types of products because they provide a good crispy texture. Insoluble fiber has been reported to either maintain or enhance the expansion of extrudates to a very limited extent, allowing for only low levels of fiber inclusion (<5% w/w) or fiber of small particle size (<125 μm) (Alam et al., 2016; Kallu et al., 2017; Wang et al., 2017).

Significant material changes occur during the extrusion process when comparing the raw inputs to the final product exiting the die, including molecular degradation, phase change, material flow, and rearrangement of molecular structures due to the shear, heat, and pressure of the extruder. Extensive studies on the behavior of starch in the extruder have been conducted for over 50 years though relatively fewer studies on fiber have been reported. Hence, the study of the behavior of starch-fiber combination is more complicated. The behavior of individual starch and fiber compositions needs to be studied so that mixed compositions can be explained to facilitate new approaches or strategies for achieving desired food qualities. However, the interaction of components within a food matrix is complex, and many have not been unveiled. Many studies have explored the effects of fiber directly on product properties (Leonard et al., 2020; Robin et al., 2012). Some studies have discussed how insoluble fiber physically affects the microstructure of extrudates (Kallu et al., 2017; Robin et al., 2010; 2011a; Wang et al., 2017), yet there are no studies discussing how fiber affects the starch matrix of extrudates at the molecular level and its relationship to microstructure and extrudate properties. Some researchers have reported that fiber is inert or rigid during extrusion processing (Camire and King, 1991), but it can disrupt the continuous phase of starch melt (Robin et al., 2011a) and rupture the cell structure of extrudates (Ganjyal et al., 2004; Wang et al., 2017). This is a likely reason why expansion is reduced with fiber inclusion. Further, the interactions between rigid insoluble fiber particles and viscoelastic starch molecules may affect how starch molecules transform and impact their expansion mechanism at the die exit, but more studies are needed to establish the explanation of these effects (Robin et al., 2012). Thus, the interactions between insoluble fiber and starch need to be further proven at the molecular level to explain their expansion mechanism.

Acceptable high-fiber products can be produced when the effects of fiber on molecular and functional changes of starch-fiber mixtures under extrusion conditions can be identified. Thus, there is a need to understand the physical and chemical changes of starch-fiber mixtures during extrusion. This information can help explain the fundamental interactions between starch and fiber, and the causes of reduced expansion. This information could further be utilized to make high-fiber expanded products with a better texture. Thus, this study aimed to investigate how the cellulose interacts physically and molecularly with the starch in the extrusion process.

## 2 Materials and methods

### 2.1 Materials

Cornstarch with 50% amylose content (S50) was obtained from Ingredion, Incorporated (Weschester, IL, USA). This cornstarch was selected because we found in our previous study that the inclusion of cellulose in cornstarch with 50% amylose content resulted in a higher expansion compared to other cornstarch types (Kallu et al., 2017). Powdered cellulose (CL) was obtained from J. Rettenmaier USA LP (Schoolcraft, MI, USA). The average particle size of the cellulose was 100 μm, as specified by the supplier. To narrow the particle size range, the cellulose was sieved using a set of screens (75, 125, 150, and 250 μm) with a Sieve Shaker (8” Sieve Tester 115V, Gilson Company, Inc., Ohio, USA). Cellulose material in the range of 75–125 μm screens was collected and used in the study. The purpose of narrowing the particle size range of cellulose was to reduce the effect of varying particle size on the expansion of extrudates. Previous studies found that particle size of fiber significantly affected the expansion of extrudates (Kallu et al., 2017; Lue et al., 1991; Wang et al., 2017).

### 2.2 Extrusion processing

Mixtures of starch and cellulose were prepared with different cellulose contents: 0% (CL0), 5% (CL5), 15% (CL15) and 30% (CL30). The blends were then mixed with distilled water using a Hobart mixer (A-200, Hobart Mfg., Troy, OH, USA) to obtain a final moisture content of 18 ± 0.5% (w.b.). All samples were stored at 4 °C overnight for moisture equilibration.

The mixtures were extruded using a co-rotating twin-screw extruder (TSE 20/40, 7.5 HP, CW Brabender, S. Hackensack, NJ, USA), with a 20-mm screw diameter and a length-to-diameter ratio (L/D) of 19.5:1. A round die (diameter of 3.15 mm) was used, and the screw profile used in this study is shown in Supplementary material (Fig. A). The temperature profile used was 50-100-140-140 °C for all experiments. Two screw speeds (150 and 250 rpm) were studied. The feeding rate was kept constant at 3.6 kg/h. The extrusion processing conditions were selected based on the literature and the preliminary trials to ensure that the studied materials could be processed within the operational ranges of the extruder. Previous studies showed that cornstarch exhibited the highest expansion at 140 °C (Chinnaswamy, 1993; Kallu et al., 2017). Extrudates were collected when the system was stabilized with constant torque and die back pressure, and then dried in a convection oven (414004-568, VWR International, LLC, PA, USA) at 45 °C for 18 h. All collected samples were stored in air-tight plastic bags at room temperature until further analysis. Extrusion experiments were conducted in triplicate. The process responses (die back pressure and motor torque), recorded by the Data Acquisition system for ATR and Intelli-Torque (CW Brabender, S.Hackensack, NJ, USA), and are presented as the average of 10 random data points during steady state operation. Specific mechanical energy (SME), the mechanical energy input per unit mass of extrudate (kJ/kg), was calculated according to Godavarti and Karwe (1997).

### 2.3 Pasting and thermal properties of raw materials

Pasting properties of raw samples were measured by a Micro Visco-Amylo-Graph (MVAG) (MVAG-U #030800LAB, CW Brabender, S. Hackensack, NJ, USA) according to Li and Ganjyal (2017) with a slight modification. Sample (10 g d.b) was mixed with 100 mL of distilled water. The temperature profile was 30 → 95 → 50 °C with the heating and cooling rates of 6 ^°^C/min, a 5-min hold at 95 °C, and a 2-min hold at 50 °C.

Thermal analysis of raw samples was performed using Differential Scanning Calorimetry (DSC) (Discovery DSC, TA Instruments, New Castle, DE, USA). Each mixture (10.0 ± 1 mg) was combined with 50 μL of distilled water in a stainless-steel sample pan (Perkin Elmer, Norwalk, CT, USA). Sample pans were then sealed with a crimper and equilibrated overnight at 4 °C. A pan with 50 μL of distilled water was used as a reference. The pans were heated from 30 to 160 °C at a rate of 5 °C/min. All tests were run in triplicate.

### 2.4 Extrudate characteristics

Expansion ratio (ER) is the ratio of the radial diameter of extrudates and die diameter. The radial diameter of extrudates was measured using a caliper (Mitutoyo America Corp., Aurora, IL, USA). For each condition, the expansion ratio of an extruded sample was recorded as the average of 20 random measurements. Water Absorption Index (WAI) and Water Solubility Index (WSI) analyses followed the methods described by Kowalski et al. (2016). Milled samples (2.5 g) were mixed with 30 mL of distilled water and then incubated for 30 min at 30 °C. Mixtures were then centrifuged at 3000 g for 10 min. The precipitated mass was weighed, and the supernatant was dried overnight. WAI (g/g) was the ratio of the precipitated mass and the initial dry mass, while WSI (%) was the percentage of the dried mass in the supernatant to that of the original sample. All analyses were performed in triplicate.

### 2.5 Microstructures of extrudates

Surface and cross-sections of selected extruded samples were cut by a precision blade. Samples were fixed on holders using conductive carbon tape. The fixed samples were sputter-coated with gold to improve their conductivity and viewed under a Scanning Electron Microscope (SEM) (FEI, Model TESCAN, FEI Company, USA) in a pressurized chamber (30 Pa) at an accelerating voltage of 30 kV. Multiple areas of a cross-section of an extruded sample were observed for overall cell structure pattern and ruptured or collapsed cell structures. Smoothness, wavy structure, or ruptured surfaces were observed for the surface of a sample. All extrudate samples were observed at magnifications of x30, x60, and x200, and raw flours and ground extrudates were observed at a magnification of x500.

### 2.6 X-ray diffraction (XRD)

X-ray diffraction spectra of raw and extruded samples were measured using an X-ray diffractometer (XRD) (MiniFlex 600, Rigaku Americas Corp., The Woodlands, TX, USA) at 40 kV and 15 mA with Cu Kα radiation. Samples were scanned over a range of 2θ from 2 to 40° at the rate of 2°/min with a step size of 0.02. Spectra data were processed using OriginLab Software (OriginLab Corp., Northampton, MA, USA.). The relative crystallinity of samples was calculated according to (Frost et al., 2009), using Eq. (1).

(1) Relative crystallinity (%) = Crystalline area/(Crystalline + amorphous area)*100

### 2.7 Fourier Transform Infrared spectroscopy (FTIR)

The molecular characteristics of starch, cellulose, their dry mixtures, and their extrudates were measured by FTIR-ATR spectroscopy (Nicolet IS10, Thermo Scientific, Waltham, MA, USA). A sample was scanned between the wavenumber range of 4000 - 600 cm^−1^ for 128 scans. The spectra resolution was 4 cm^−1^. All spectra were analyzed using Omnic Sofware, the built-in program of Nicolet iS10. The spectra were baseline corrected by drawing a straight line between 1200 and 800 cm^−1^. The absorbance intensity at 995, 1015, and 1045 cm^−1^ from the baseline was collected for each sample. The ratio of the absorbance intensities: 1045/1015 cm^−1^ and 995/1015 cm^−1^ were calculated for the comparison of short-range conformation of the samples as described in (Shrestha et al., 2010; Warren et al., 2016).

### 2.8 Statistical analysis

Analysis of Variance (ANOVA) was used to analyze the mean differences between the extrusion process responses and extrudate characteristics. The normality of data was assessed by the Shapiro-Wilk test, and the comparison of means was assessed by Tukey's test with the defined significance of 5% (*P* < 0.05). The Pearson correlation coefficient analysis between cellulose content, crystallinity, FTIR peak ratio, and the physical properties of extrudates was also performed. These analyses were conducted using SPSS software (IBM SPSS® Statistics®, SPSS Inc., Chicago, USA).

## 3 Results and discussions

### 3.1 Pasting and thermal properties of starch-cellulose mixtures

Table 1 shows the pasting properties of raw mixtures. Raw starch had a pasting temperature (T_paste_) of 89 °C, while cellulose did not have a T_paste_. Cellulose is insoluble, and it was not disrupted to a large degree by the temperature in MVAG. As the cellulose content increased, T_paste_ of the mixtures increased from 89.3 °C to 92.7 °C, and their peak viscosity (PV) and final viscosity (FV) decreased. The peak viscosity of the sample with 15% cellulose was reduced by almost half compared to the pure starch. The decrease of PV due to increasing fiber content was also reported in the starch-sugarcane bagasse study (Masli et al., 2018).

**Table 1:** Pasting and thermal properties of raw starch-cellulose mixtures.

CL (g/kg)	Pasting properties			Thermal properties			
	T_paste_ (^o^C)	PV (mPa.s)	FV (mPa.s)	T_o_ (^o^C)	T_p_ (^o^C)	T_c_ (^o^C)	ΔH (J/g)
0	89.3 ± 0.5^c^	68.0 ± 1.0^a^	62.3 ± 1.5^a^	70.6 ± 0.3^a^	83.5 ± 0.1^a^	107.8 ± 0.9^a^	10.6 ± 0.3^a^
50	90.1 ± 0.5^c^	54.0 ± 2.0^b^	52.0 ± 2.0^b^	69.7 ± 0.5^a^	84.4 ± 1.9^a^	106.4 ± 0.4^a^	10.7 ± 0.2^a^
150	91.1 ± 0.1^b^	40.3 ± 1.2^c^	39.7 ± 0.6^c^	70.6 ± 0.1^a^	83.4 ± 0.2^a^	106.2 ± 0.2^a^	8.5 ± 0.3^b^
300	92.7 ± 0.3^a^	25.3 ± 0.6^d^	26.3 ± 0.6^d^	70.6 ± 0.6^a^	84.7 ± 0.8^a^	107.9 ± 1.2^a^	7.6 ± 0.2^c^
Cellulose	ND	13.6 ± 0.6^e^	14.3 ± 0.6^e^	ND	ND	ND	ND

All values are Mean ± SD (n = 3). Values with different letters within a column indicate a significant difference between means (*P* < 0.05). CL: Cellulose content, T_paste_: Pasting Temperature, PV: Peak viscosity, FV: Final viscosity **T**_**o**_: Onset temperature, **T**_**p**_: Peak temperature, **T**_**c:**_ Concluding temperature, **ΔH**: Enthalpy changes, ND: Not Detected.

The thermal properties of the raw samples are shown in Table 1. There was a statistically insignificant increase of onset and peak melting temperature (T_o_ and T_p_) as the cellulose content increased (P > 0.05). However, the melting enthalpy decreased as cellulose content increased. It could be attributed to the pure cellulose not undergoing any phase changes at these conditions (Supplementary material Fig. B1). Therefore, the reduced enthalpy is due to the reduced amount of starch in the mixture and the absence of a phase change of cellulose.

These results suggested that cellulose appeared to influence the extent of starch gelatinization as a function of the reduced enthalpy and the increased pasting temperature, but cellulose did not significantly affect the melting temperature of starch.

### 3.2 Extrusion characteristics

#### 3.2.1 Process responses

Table 2 shows the torque, pressure, and specific mechanical energy of the extrudates of starch-cellulose mixtures. Torque decreased as the screw speed increased, which is the general trend observed for the extrusion of starches (Kaisangsri et al., 2019; Masli et al., 2018). For each screw speed, the torque values were not significantly different when cellulose content increased to 15%. At 30% of cellulose, the torque was 22.3 ± 0.9 Nm, which slightly decreased compared to the other three samples. Similar observations were made for the values of back pressure and SME. It could be attributed to the different nature of starch and cellulose, as it can be seen in their pasting and thermal properties (Section 3.1). Under shear, heat with the presence of a sufficient amount of water, starch swells and gelatinizes, and transforms to paste (with a certain viscosity). On the contrary, cellulose does not swell and transform to paste because cellulose is not disrupted to any great extent due to its resistant molecular structures. Therefore, at a certain cellulose content in the starch-cellulose mixtures, the paste-like viscosity significantly reduced and disrupted by the cellulose, leading to phase separation (phase 1: paste-like of starch and phase 2: small particles of cellulose), as inferred by torque and pressure values.

**Table 2:** Extrusion characteristics of extruded starch-cellulose mixtures at different cellulose contents.

Sample code	CL (g/kg)	SS (rpm)	T (Nm)	P (MPa)	SME (kJ/kg)	ER	WAI (g/g)	WSI (%)	Ratio 995/1015	Ratio 1045/1015
**CL0-1**	**0**	**150**	23.4 ± 0.7^a^	6.7 ± 0.2^ab^	392.3 ± 12.4^d^	3.3 ± 0.1^b^	3.1 ± 0.2^a^	15.9 ± 1.8^de^	1.06 ± 0.0^a^	0.52 ± 0.0^c^
**CL5-1**	**50**		23.3 ± 0.6^a^	6.8 ± 0.1^a^	391.8 ± 10.7^d^	3.2 ± 0.1^b^	3.0 ± 0.2^a^	16.9 ± 0.5^cd^	1.06 ± 0.0^a^	0.52 ± 0.0^c^
**CL15**–**1**	**150**		23.0 ± 1.1^a^	6.6 ± 0.3^b^	385.7 ± 17.9^de^	2.5 ± 0.0^c^	2.8 ± 0.2^b^	16.7 ± 0.6^d^	1.05 ± 0.0^b^	0.52 ± 0.0^c^
**CL30**–**1**	**300**		22.3 ± 0.9^b^	6.4 ± 0.2^c^	375.1 ± 15.7^e^	1.9 ± 0.1^d^	2.2 ± 0.0^c^	15.1 ± 0.8^e^	1.03 ± 0.0^d^	0.56 ± 0.0^b^
**CL0-2**	**0**	**250**	17.5 ± 0.5^c^	5.4 ± 0.1^d^	490.1 ± 14.7^a^	3.6 ± 0.1^a^	3.2 ± 0.2^a^	18.5 ± 0.5^bc^	1.05 ± 0.0^b^	0.52 ± 0.0^c^
**CL5-2**	**50**		17.3 ± 0.6^cd^	5.4 ± 0.1^d^	483.4 ± 17.6^a^	3.2 ± 0.1^b^	3.0 ± 0.2^a^	20.4 ± 1.5^a^	1.05 ± 0.0^b^	0.52 ± 0.0^c^
**CL15**–**2**	**150**		17.1 ± 0.6^cd^	5.3 ± 0.2^f^	478.9 ± 17.2^bc^	2.6 ± 0.0^c^	2.7 ± 0.1^b^	19.5 ± 1.4^ab^	1.04 ± 0.0^c^	0.53 ± 0.0^bc^
**CL30**–**2**	**300**		16.8 ± 0.4^d^	5.4 ± 0.1^df^	470.9 ± 9.8^c^	1.9 ± 0.0^d^	2.2 ± 0.1^c^	17.1 ± 0.7^cd^	1.03 ± 0.0^d^	0.59 ± 0.0^a^

All values are mean ± SD (30 ≥ n ≥ 9); values with different letters within a column indicate a significant difference between means (*P* < 0.05). CL: Cellulose content, SS: Screw Speed, T: Torque, P: Pressure, SME: Specific Mechanical Energy, ER: Expansion Ratio, WAI: Water Absorption Index, WSI: Water Solubility Index. Ratio (995/1015) and (1045/1015): FTIR peak ratios (the absorbance intensity of the peaks at 995, 1015 and 1045 cm^−1^).

#### 3.2.2 Extrudate characteristics

Despite the similar values observed for the process responses, the expansion ratio (ER) of extrudates reduced from 3.3 ± 0.1 to 1.9 ± 0.1 at 150 rpm, and from 3.6 ± 0.1 to 1.9 ± 0.0 at 250 rpm, as the cellulose content increased from 0% to 30%, respectively (Table 2). The ER of starch (CL0) and 5%-cellulose extrudate (CL5) were not significantly different at a screw speed of 150 rpm. This could be due to the relatively small amount and/or small particle size of cellulose, which enabled it to be well dispersed within the starch melt phase. However, as the cellulose level increased, its contribution to the disruption of the starch phase became more prominent, and the friction increased as starch-cellulose domains are more likely to overlap, leading to a reduction in ER. Interestingly, the ER for each different cellulose content at different screw speeds was not significantly different, while for extrudates of only starch (without any cellulose), ER increased with increasing screw speed. This result suggests that cellulose may be resistant to breakdown under high shear (high screw speed) and may interfere with the degradation of starch by the shear during extrusion. Given that the process responses were similar, cellulose may interfere at the molecular level with the ability of starch molecules to reassociate after exiting the die, that is, as it is transformed from the viscoelastic phase to the glassy phase (Ek et al., 2020; Núñez et al., 2009). During the expansion phenomena in extrusion, low viscosity and low elasticity of the mixtures affected by the cellulose could be the reason for low expansion rather than the change of melting temperature. This supported the previous findings that the fiber disrupted the continuous phase of starch (Ganjyal et al., 2004; Robin et al., 2011a).

Water Absorption Index (WAI) also showed a similar trend as ER. It decreased as the cellulose content increased but was not significantly different between 150 rpm and 250 rpm for each of the cellulose levels. This result is in agreement with several previous reports that concluded, increasing fiber contents decreased WAI of extrudates (Robin et al., 2011b; Wang et al., 2017). In contrast, Kallu et al. (2017) reported that the WAI of 50%-amylose starch with cellulose increased with increasing cellulose content up to 10% (w/w). Interestingly, some studies reported that WAI of extrudates either increases or decreases with the increase of fiber content, depending on the extrusion processing conditions and the resulting expansion ratio (da Silva Alves et al., 2018; Kaisangsri et al., 2016). Therefore, WAI of extrudates could be influenced by the type and level of starches and fibers, the impacts of extrusion conditions, the degree of starch gelatinization during extrusion, and the expansion property of extrudates.

WSI increased as the expansion ratio increased because of the degradation of starch. WSI slightly increased at 5% and 15% (w/w) cellulose contents and then decreased at 30% (w/w) of cellulose (Table 2). Kallu et al. (2017) also reported the increased WSI of extrudates with 0%–10% (w/w) cellulose content. Meanwhile, the studies on the inclusions of wheat bran, carrot pomace, cherry pomace in extrudates found that the WSI decreased with increasing fiber contents, notably at the fiber content >5% (w/w) (Kaisangsri et al., 2016; Robin et al., 2011b; Wang et al., 2017). It could be due to the interaction effects of reduced starch content and increased cellulose content in the mixtures (Dey et al., 2021). At low cellulose contents (up to 15%, w/w), mechanical shear during extrusion could be sufficient to breakdown cornstarch and transform some cellulose molecules to be more soluble. At a cellulose content >15%, there was a lesser amount of starch and more cellulose that likely prevented their degradation due to shear during extrusion. A similar explanation was given in a study of wheat bran and wheat flour that WSI of extrudates is positively correlated with mechanical shear at low wheat bran content, while this correlation is weak at the high content of wheat bran (Robin et al., 2011b).

### 3.3 Microstructures of extrudates

**Fig. 1:**
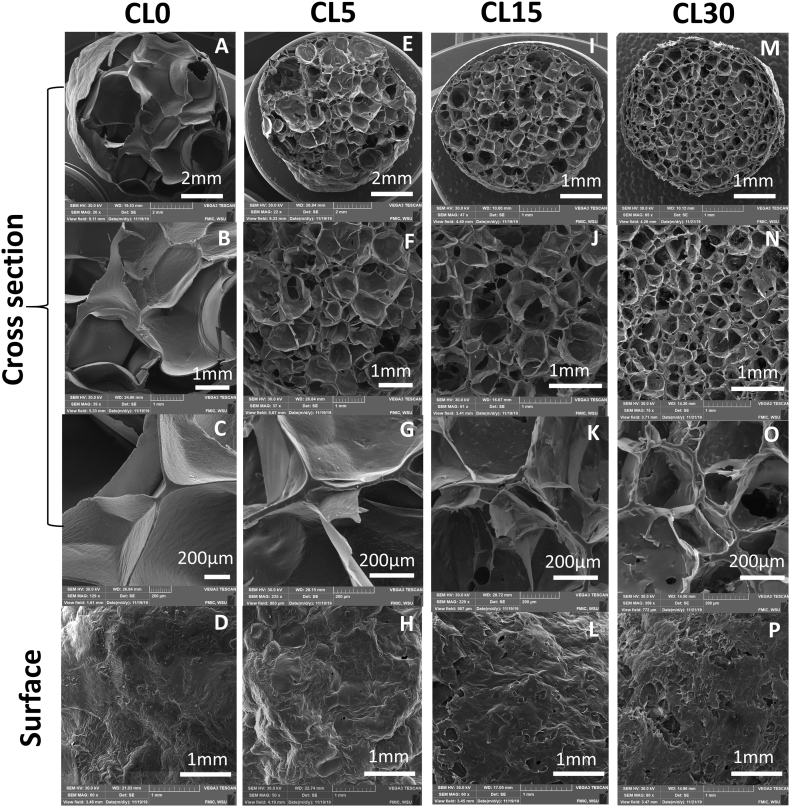
Structure of extrudates by Scanning Electron Microsocpe (SEM) at 250 rpm. CL0, CL5, CL15 and CL30 represents the extrudates with 0%, 5%, 15% and 30% cellulose, respectively.

**Fig. 2:**
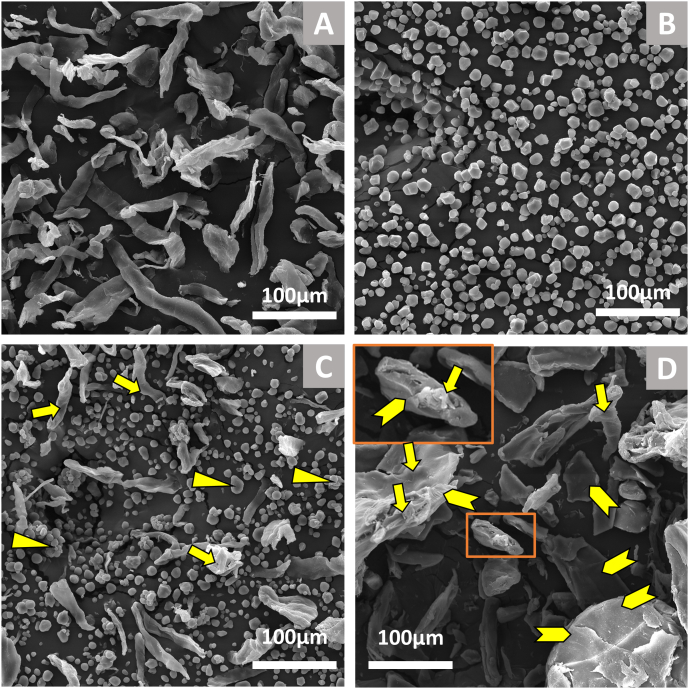
The microstructure of raw cellulose (A), raw starch (B), and starch-cellulose mixture (CL30, 30% cellulose content): raw flour (C) and ground extrudate (D) by Scanning Electron Microscope (SEM). Note:  Cellulose;  Starch;  gelatinized starch;  An example of fiber entrapped in gelatinized starch.

Fig. 1 shows the micrographs of cross-sections and surfaces of starch-cellulose extrudates at different magnifications. For cross-sections, as cellulose content increased, the diameter and the cell sizes of extrudates decreased. Cell sizes of the extrudates became more uniform with increasing cellulose content. Fiber can act as a physical nucleating agent, which increases nucleating sites for bubble growth during an expansion (Bénézet et al., 2012; Robin et al., 2011a). In this case, cellulose could act as a filler within the cell walls of extrudates, or it may be dispersed and interact with starch to create bubble cells in extrudates. However, as cellulose content increased, the cell walls became thinner and more ruptured (Fig. 1 C, G, K, and O). The surface of extrudates with a 30% cellulose content showed significant surface disruption. It could be due to the increased friction when the melt flowed through the die. Fig. 2 shows raw starch, raw cellulose, the raw mixture, and ground extrudate of CL30. By comparing the raw and extruded flour, as indicated by the arrows in Fig. 2C and D, starch granules gelatinized and transformed into compact solid, while the microstructure of cellulose remained similar, and some of them are covered by gelatinized starch. It could indicate a likely phase separation between cellulose and starch during extrusion. The processing of bio-composites with different starches and fibers showed that at a certain starch/cellulose ratio, cellulose could not be well dispersed in starch matrix, leading to the agglomerations of cellulose in the continuous starch phase because of poor interfacial adhesion between cellulose and starch matrix (Chen et al., 2020; Ghanbari et al., 2018; Hietala et al., 2013). Miscibility and compatibility between starches and fibers depends on their types and the types of plasticizers utilized (Kibar and Us, 2017; Liu et al., 2017; Lomelí-Ramírez et al., 2014). Therefore, if interfacial adhesion between cellulose and starch matrix is poor and the amount of hydrogen bonding of cellulose, starch, and plasticizer is not sufficient, then phase separation can occur (Kibar and Us, 2017; Liu and Budtova, 2012).

### 3.4 Long-range molecular changes (XRD)

**Fig. 3:**
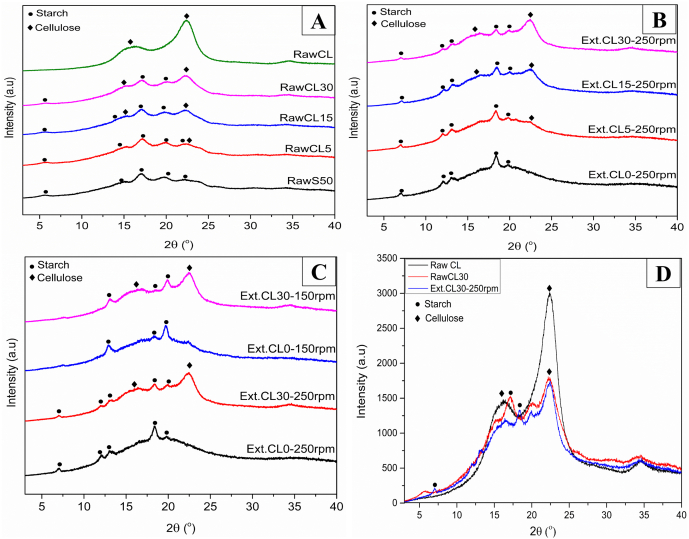
XRD spectra: (A) Raw starch-cellulose mixtures; (B) Extrudates with different cellulose contents, extruded at 250 rpm; (C) Extrudates extruded at 150 rpm and 250 rpm; (D) Comparison of raw and extrudate of starch-cellulose mixtures. Raw S50 (Raw starch), Raw CL (Raw cellulose), Ext. (Extrudates), CL (Cellulose), at 0, 5, 15 and 30% of cellulose content.

XRD diffractograms show the changes in the crystalline structure of raw and extruded mixtures. Fig. 3 shows the XRD diffractograms of raw starch, cellulose, and the extrudates of their mixtures. Depending on their botanical origin and amylose content, starch granules exhibit three main types of X-ray diffraction patterns corresponding with crystalline polymorphic forms: A-type for cereal starches, B-type for tubers and high amylose starches, and C-type (coexisting of A and B crystals in the granule) for leguminous starches (Lopez-Rubio et al., 2008). In addition, V-type crystallinity has been described as the native helical arrangement from single amylose helices or its complex with endogenous granular lipid, or as the new helical arrangement of single helices from disrupted starch molecules during gelatinization, associating with the starch retrogradation (Frost et al., 2009; Lopez-Rubio et al., 2008). The XRD pattern of raw starch (Fig. 3A) exhibited B-type crystallinity with major peaks at 5.5°, 15.26°, 17.21°, 19.75°, and 22.32°, which is generally observed in cornstarch with high amylose content (Pozo et al., 2018; Shrestha et al., 2015). Raw cellulose had the typical XRD pattern for cellulose I, with crystalline peaks at 16.1° and 22.1° (Lu et al., 2013). For the raw mixtures, the intensity of peak 22.1° increased with increasing cellulose content; thus, this peak explained the presence of cellulose in the mixtures.

In Fig. 3B, the XRD spectra of the extruded starch sample (Ext.CL0) had a sharp and high intensity peak at 18.4°. The intensity of the peak at 17.21° became broader and less intense, while that at 18.4° and 19.75° became sharper, suggesting the loss of B-type crystallinity and an increase of V-type crystallinity (Shrestha et al., 2015). In Fig. 3C, the samples extruded at 250 rpm had the increased intensity of the peaks at 7°, 13°, and 18.4° compared to the extrudates at 150 rpm, which had an increased peak at 14° and 19.75°. These long-range molecular changes suggest that more V-type crystallinity of a single helix was formed at higher mechanical shear. For the extruded mixtures with 30% cellulose (CL30), the peaks representing starch remained relatively the same, and the peak (22.1°) representing cellulose increased as the cellulose content increased. By comparing the XRD spectra of raw CL30 and its extrudate in Fig. 3D, the intensity of peak at 22.1° remained the same, suggesting that cellulose did not undergo long-range molecular changes while starch did.

Table 3 shows that for all raw and extruded starch-cellulose mixtures, the crystallinity increased with increasing cellulose content. After extrusion, the crystallinity decreased compared to the raw mixtures, as expected, because starch was gelatinized and had a less ordered structure. Lower crystallinity values were observed for the extrudates extruded at 250 rpm as high screw speed provided high mechanical shear to disrupt the ordered structure of the materials.

**Table 3:** Relative crystallinity of raw and extruded starch-cellulose mixtures.

CL (g/kg)	Crystallinity (%)		
	Raw	After extrusion at 150 rpm	After extrusion at 250 rpm
0	19.1 ± 0.5^e^	9.4 ± 0.8^d^	8.9 ± 0.2^c^
50	22.1 ± 1.8^d^	12.4 ± 0.9^c^	10.0 ± 0.8^c^
150	27.2 ± 1.1^c^	17.0 ± 0.8^b^	13.7 ± 1.7^b^
300	28.8 ± 0.6^b^	21.8 ± 1.6^a^	20.2 ± 1.5^a^
Cellulose	61.8 ± 2.0^a^	NA	NA

All values are mean ± SD (n = 3); values with different letters within a column indicate a significant difference between means (*P* < 0.05). CL: Cellulose content.

### 3.5 Short-range molecular changes (FTIR)

**Fig. 4:**
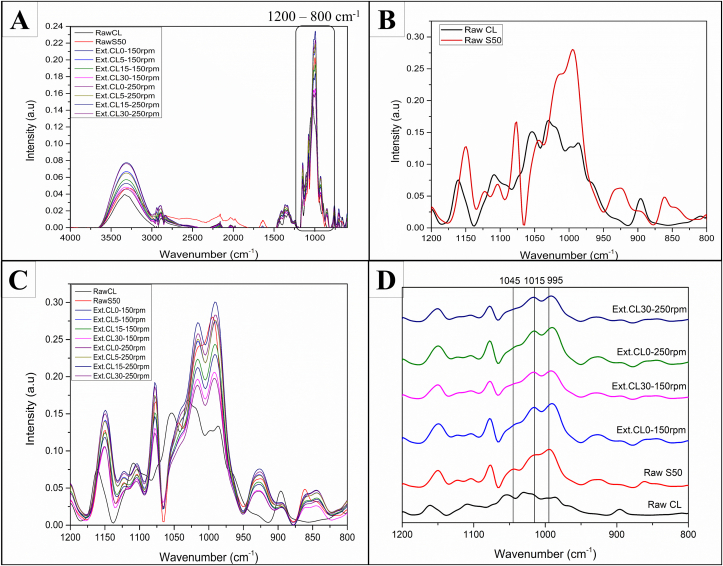
FTIR spectra: (A) raw and extruded samples at the wavenumber range 4000-600 cm^−1^ (B) raw starch (raw S50) and raw cellulose (raw CL) at fingerprint region 1200-800 cm^−1^ (C) raw and extruded samples at the fingerprint region (D) select raw samples and extruded samples for peak comparisons. Ext. (Extrudates), CL (Cellulose), at 0, 5, 15 and 30% of cellulose content.

FTIR spectra provide information on the chemical functionality and short-range order of molecules. Fig. 4 shows FTIR spectra for raw and extruded products. Fig. 4A provides the comparison of all the FTIR spectra in the range of wavenumbers 4000-600 cm^−1^. Band assignments for raw starch and cellulose are shown in the supplementary material (Fig. C.1 and Table C.1 and C.2).

Peaks of all spectra exhibited similar absorbance bands with different intensities. No new peaks were observed, which could mean that no new covalent bonds or functional groups had been formed (Yin et al., 2020). The molecular changes before and after extrusion could be dominated by the cleavage of glycosidic linkages and the formation of hydrogen bonds, which change the conformations of the molecular structures of the material during the melting in the extruder and the reassociation of chains after exiting the die. For this reason, cellulose may interfere with how starch chains re-associate to make new ordered structures and alter their structural conformation at the die exit to transform the material into a glassy state. More importantly, the presence of cellulose could affect the hydrogen bonding and crystallinity changes in the starch matrix. The studies on bio-composite production by extrusion also reported that no new peaks in FTIR were observed in the bio-composites of starch/microcrystalline cellulose, or starch/soybean hulls (Chen et al., 2020; Merci et al., 2019). The authors concluded that the molecular interaction between starch and fiber is mainly hydrogen bonding.

There are a few different absorbance bands in the region of 1200-800 cm^−1^ (Fig. 4B, C, and D), which depict C–O, C–C, C–O–H stretching, and C–O–H bending. This region has also been observed to be sensitive to the molecular changes in starch structure (Capron et al., 2007; Warren et al., 2016). Fig. 4B shows the spectra for raw cellulose and raw starch. Raw starch and cellulose have different absorbance bands due to their different bond configurations and structural conformations, even though they are both made of D-glucose monomers.

Fig. 4C shows the comparison of spectra of both extrudates and the raw samples. The FTIR spectra of the mixtures had the same patterns as the cornstarch, except that they had different intensities, which was attributed to a high amount of starch in the mixtures.

Intensities of the absorption for all extruded samples decreased as fiber content and screw speed increased compared to raw starch samples. Shrestha et al. (2010) observed that FTIR spectra for all starches extruded at different feed moisture contents had peaks at the same positions and almost the same intensities compared to the raw starch. Thus, the decrease in the intensities of FTIR spectra with increasing cellulose content could be attributed to the absorbance by molecular bonds of cellulose, which made the signals of the peaks lower as there was an overlapping of peaks. It could also be attributed to the reduced amount of starch in the mixtures and the destruction of the covalent bonds (glycosidic bonds) within starch after extrusion.

There were notable changes in peaks 1045, 1015, and 995 cm^−1^, which have been described to be sensitive to molecular changes in starch structure (Capron et al., 2007; Warren et al., 2016). These three peaks have been assigned to C–OH bending and the C–H_2_ related modes (van Soest et al., 1995). Peak 1045 cm^−1^ is associated with a crystalline form of starch, and it decreases during gelatinization and increases during retrogradation (Sevenou et al., 2002). Peak 1015 cm^−1^ is associated with amorphous starch. Its intensity increases with the loss of ordered structure (from native to gelatinized starch) and decreases during reordering (from gelatinized to retrograded starch). Peak 995 cm^−1^ is also associated with the crystalline structure, especially for native starch, and is sensitive to water content and related to intramolecular hydrogen bonding (Van Soest and Vliegenthart, 1997). The changes or shifts in this peak suggest the modification of hydrogen bonding of double helices (Van Soest and Vliegenthart, 1997). Peaks 1045 and 1015 cm^−1^ represent the C–OH bending modes and are associated with ordered and amorphous starch, respectively (Capron et al., 2007; van Soest et al., 1995).

To clearly illustrate the changes in these peaks, Fig. 4D depicts the selected spectra in a stacked format. The intensity of the peak at 1045 cm^−1^ was reduced for all the extruded samples, while the intensity of 1015 cm^−1^ increased after extrusion relative to the intensity of 995 cm^−1^ for all treatments. In addition, the peak at 995 cm^−1^ of the extrudates containing 30% cellulose (CL30) was wider compared to the extrudates without cellulose (CL0). This peak is sensitive to structural changes and is associated with hydrogen bonding; thus, the result suggests the interference of cellulose with starch reassociation (retrogradation) in the extrudates.

### 3.6 Relationship between crystallinity, FTIR peaks ratios, and physical properties of extrudates

Ratios of the peaks in FTIR spectra, 995/1015 and 1045/1015 (995/1022, 1047/1022 in some previous studies), have been studied as indexes for the short range order of molecules, indicating the degree of order in starch molecules (Shrestha et al., 2010; Warren et al., 2016). In this study, the peak ratio of 995/1015 cm^−1^ decreased after extrusion (1.17 for raw starch to 1.06-1.03 for extrudates), which explained the destruction of native starch order structure after extrusion. The XRD data supported this result because the crystallinity of all samples reduced after extrusion. Capron et al. (2007) also reported that raw starch has higher ratios of 1000/1022 cm^−1^ (995/1015 cm^−1^ in this study) compared to the extruded starch.

However, Table 2 showed that the peak ratio 995/1015 cm^−1^ of extrudates decreased as the cellulose content increased. Also, Table 4 shows that peak ratio 995/1015 cm^−1^ of starch-cellulose extrudates had a strongly negative correlation with the crystallinity of extrudates (r = −0.831, P < 0.01). This result is contradicted with the previous studies on extruded starch alone (Capron et al., 2007; Shrestha et al., 2010), which could mean peak ratio 995/1015 cm^−1^ cannot explain the crystallinity as the case of starch alone. It is important to note that after the extrusion, the crystallinity decreased, and XRD diffractogram revealed that V-type crystallinity was formed, and it is likely to be more at higher mechanical shear. Similar results were reported by (Shrestha et al., 2010).

**Table 4:** Pearson’s correlation matrix of cellulose content, crystallinity (XRD), FTIR peak ratios, specific mechanical energy, and physical properties of extrudates.

	CL	Crystallinity (XRD)	Ratio (995/1015)	Ratio (1045/1015)	SME	ER	WAI	WSI
CL	1							
Crystallinity (XRD)	0.953^b^	1						
Ratio (995/1015)	-0.917^b^	-0.831^b^	1					
Ratio (1045/1015)	0.423^a^	0.472^a^	-0.438^a^	1				
SME	-0.140	-0.327	0.009	0.050	1			
ER	-0.983^b^	-0.946^b^	0.875^b^	-0.398	0.235	1		
WAI	-0.972^b^	-0.907^b^	0.906^b^	-0.424^a^	0.082	0.948^b^	1	
WSI	-0.366	-0.512^a^	0.330	-0.218	0.805^b^	0.417^b^	0.341	1

CL: Cellulose content, Ratio (995/1015) and (1045/1015): FTIR peak ratios (the absorbance intensity of the peaks at 995, 1015 and 1045 cm^-1^).

a Correlation is significant at the 0.05 level.

b Correlation is significant at the 0.01 level (2-tailed).

The peak ratio 1045/1015 cm^−1^ decreased from 0.57 (raw starch) to 0.52 (extruded starch), but this ratio for the extruded samples increased as the cellulose content increased (Table 2). There was a positive correlation between peak ratio 1045/1015 cm^−1^ and crystallinity (r = 0.472, P < 0.05). Warren et al. (2016) reported a weak correlation between the peak ratio 1045/1015 cm^−1^ and the ordered structure of starch by XRD. Therefore, this peak ratio may be of less interest compared to the peak ratio 995/1015 cm^−1^ to study the molecular changes in starch and cellulose by FTIR.

ER was strongly negatively correlated to crystallinity (r = −0.946, P < 0.01), which means increasing crystallinity of the materials decreased the ER of extrudates. SME also had a negative correlation with crystallinity, even though the correlation was not significant. In general, SME affected the degree of starch degradation (Kowalski et al., 2018). However, with the presence of cellulose, SME had less effect on material degradation because cellulose is more resistant to the shear.

## 4 Conclusions

The presence of cellulose significantly affected the physicochemical properties of extrudates. Increased cellulose content reduced the expansion ratio of extrudates with smaller porous cell structures. Peak melting temperatures of materials remained unchanged with the increasing cellulose inclusion, suggesting that cellulose is resistant to breakdown and did not go through any phase change under the conditions studied. Results from FTIR suggested that no covalent bonding interaction between starch, fiber, and water existed under extrusion conditions but that their interactions are mainly due to the hydrogen bonding. Further, fiber may interfere with how starch biopolymers reassociate through intermolecular hydrogen bonds. XRD results supported that cellulose did not undergo a great extent of molecular changes as starch did. The effects of cellulose on physicochemical properties could mainly be attributed to a likely phase separation between starch and cellulose in the extruder (phase 1: paste-like of starch and phase 2: small particles of cellulose) and the disruption of intermolecular hydrogen bonding of starch by cellulose during the expansion at the die exit. Further studies of extrusion with higher cellulose content greater than 30% may help to explain clearly the structural and molecular changes of cellulose. Also, the rheological studies of starch-cellulose mixtures may be useful for explaining the phase separation phenomenon. At the microstructural level, color staining microscopic methods such as confocal microscopy should be performed to confirm that cellulose fiber acts as a filler within the cell walls of extrudates and/or interfere with starch melt to form cells of extrudates.

## CRediT authorship contribution statement

**Pichmony Ek:** Conceptualization, Methodology, Formal analysis, Investigation, Writing – original draft, Visualization. **Bon-Jae Gu:** Methodology, Investigation, Writing – review & editing. **Steven R. Saunders:** Writing – review & editing. **Kerry Huber:** Writing – review & editing. **Girish M. Ganjyal:** Conceptualization, Writing – review & editing, Supervision, Project administration, Funding acquisition.

## Declaration of competing interest

The authors declare that they have no known competing financial interests or personal relationships that could have appeared to influence the work reported in this paper.
