# Supplementary material for: Exploration of physicochemical properties and molecular interactions between cellulose and high-amylose cornstarch during extrusion processing
Source: Curr Res Food Sci. 2021 Jul 8;4:588–97. doi: 10.1016/j.crfs.2021.07.001 (PMC8405956; doi:10.1016/j.crfs.2021.07.001)
Supplement: Multimedia component 1 [file mmc1.docx]

**Supplementary data**

**Exploration of physicochemical properties and molecular interactions between cellulose** Pichmony Ek^a,c^, Bon-Jae Gu^a^, Stevens S. Saunders^b^, Kerry Huber^d^ , Girish M. Ganjyal^a*^

^a^ School of Food Science, Washington State University, Pullman, WA 99164 -6376 (USA)

^b^The Gene and Linda Voiland School of Chemical Engineering and Bioengineering,

Washington State University, Pullman, WA, 99164-6515 (USA)

^c^Faculty of Chemical and Food Engineering, Institute of Technology of Cambodia, Phnom Penh, Cambodia.

^d^Department of Animal and Food Science, Brigham Young University-Idaho, Rexburg, ID, 83460 (USA)

*Corresponding Author:

Girish M. Ganjyal

School of Food Science

P.O. Box 646376

Washington State University

Pullman, WA 99164-6376

Phone: +001-509-335-5613

Fax: +001-509-335-4815

Email: [girish.ganjyal@wsu.edu](mailto:girish.ganjyal@wsu.edu)


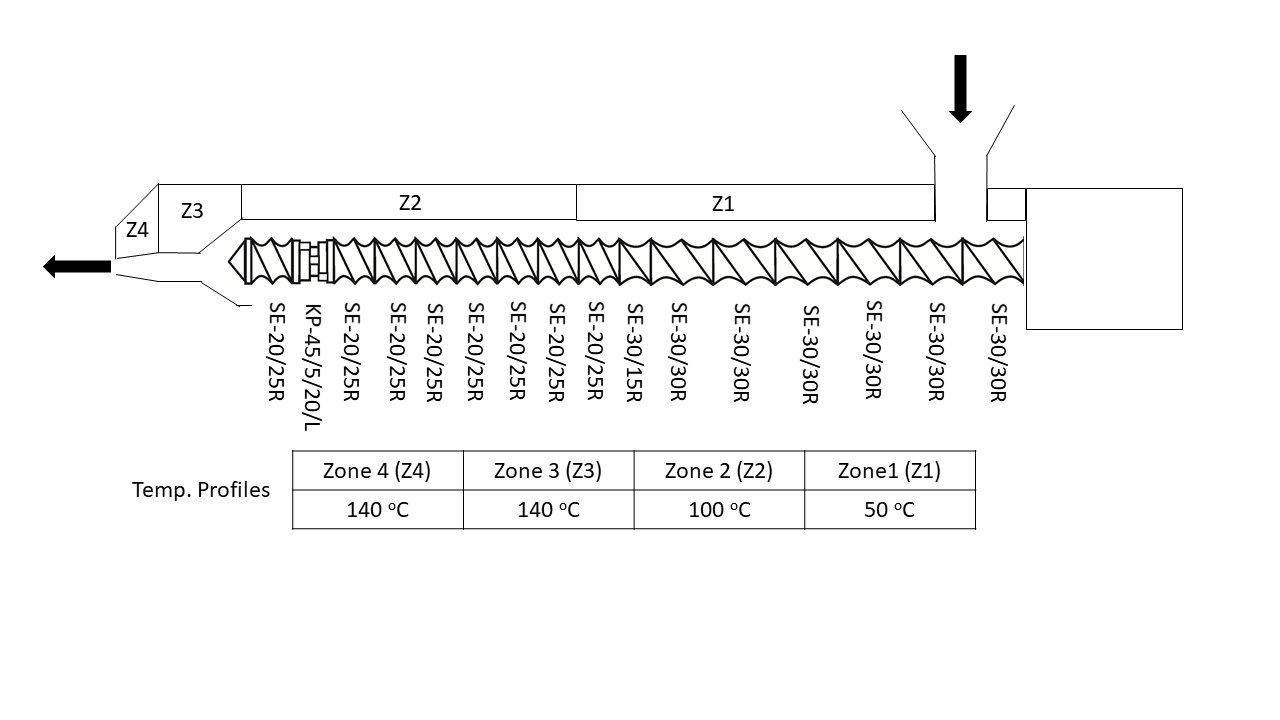


**Figure A.** Screw configuration and temperature profile of the extruder used in this study.

**Figure B.** Pasting and thermal properties of raw starch-cellulose mixtures: (B1) Pasting profile by Micro-ViscoAmylograph (MVAG); (B2) Thermogram by Differential Scanning Calorimetry (DSC). RawCL100: Raw Cellulose; RawCL0: raw starch; RawCL5, CL15, CL30: raw starch-cellulose mixture with 5, 15, and 30% of cellulose content.







**D**

**C**







**Figure C.1**. FTIR of raw starch (50% amylose) and cellulose

**Table C.1** Band assignments of starch

| **Wavenumber (cm^-1^)** | **Band assignments/Association to molecular changes of starch** |
| --- | --- |
| 3280 | O - H bond stretching mode of water |
| 2888 | O-H intramolecular bond |
| 1641 | C=O, -OH bending mode of water |
| 1335 | C-H bending, O-H in-plane bend, O-C-H bending, C-C-H, C-O-H |
| 1200-800 region | C-C; C-O; with some C-OH bending  Sensitive to changes in starch |
| 1125 | CO, CC stretching, with some C-OH contributions |
| 1150 | C-OH stretching |
| 1103 | CO, CC stretching, with some C-OH contributions |
| 1077 | C-OH bending, C-H2 related modes |
| **1045 (1047)** | C-OH bending, C-H2 related modes  Linked to the degree of order/crystallinity in starch molecules  Decrease during gelatinization, increase during retrogradations |
| **1015 (1022)** | C-OH bending, C-H2 related modes, C-H stretching modes in alcoholic COH moieties  Linked to the amorphous form of starch, Increase with decreasing crystallinity |
| **995** | C-OH bending, C-H2 related modes, C-H stretching modes in alcoholic COH moieties  Linked to the degree of order/crystallinity  Unclearly recognized as water sensitive and related to intramolecular hydrogen bonding of hydroxyl groups, the plasticizing effect of water (hydrogen bonding of OH of 6), change of this peak means hydrogen bonding of double helice had significant modification. |
| 926 | C-OH bending, C-H2 related modes, Glycosidic bonds (950-900) |
| 861 | C-OC (COC symmetrical stretching) |
| **995/1015 (995/1022)** | Degree of crystallinity, Decrease with decreasing crystallinity |
| **1045/1015 (1047/1022)** | Degree of crystallinity  Decrease with decreasing crystallinity |

**References:** Capron et al., 2007; Sevenou et al., 2002; Shrestha et al., 2010; Van Soest et al., 1995; Warren et al., 2013

**Table C.2** Band assignments of cellulose

| **Wave number (cm^-1^)** | **Band assignments/Association to molecular changes of Cellulose** |
| --- | --- |
| 3325 | -OH, can be related to dislocation regions (3450-3264 cm-1)  OH stretching intramolecular hydrogen bond |
| 2884 | C-H symmetrical stretching |
| 2161 |  |
| 1316 | C-H bending in plane (1368-1360) |
| 1200-800 (region) |  |
| 1161 | C-OC glycoside ether band |
| 1109 |  |
| 1054 | C-O-C pyranose ring stretching vibration |
| 1030 | C-C, C-OH, C-H ring, and side group vibration, C-O stretching vibration (2) |
| 987 | C-H aromatic hydrogen |
| 896 | C-O-C, C-C-O, C-CH deformation and stretching, β-glycosidic absorption group ??? Glycosidic-C1H deformation (2) |

References: Melikoğlu et al., 2019; Szymanska-Chargot et al., 2017

**References**

Capron, I., Robert, P., Colonna, P., Brogly, M., & Planchot, V. (2007). Starch in rubbery and glassy states by FTIR spectroscopy. *Carbohydrate Polymers*, *68*(2), 249–259. https://doi.org/10.1016/j.carbpol.2006.12.015

Melikoğlu, A. Y., Bilek, S. E., & Cesur, S. (2019). Optimum alkaline treatment parameters for the extraction of cellulose and production of cellulose nanocrystals from apple pomace. *Carbohydrate Polymers*, *215*(January), 330–337. https://doi.org/10.1016/j.carbpol.2019.03.103

Sevenou, O., Hill, S. E., Farhat, I. A., & Mitchell, J. R. (2002). Organisation of the external region of the starch granule as determined by infrared spectroscopy. *International Journal of Biological Macromolecules*, *31*(1–3), 79–85. https://doi.org/10.1016/S0141-8130(02)00067-3

Shrestha, A. K., Ng, C. S., Lopez-Rubio, A., Blazek, J., Gilbert, E. P., & Gidley, M. J. (2010). Enzyme resistance and structural organization in extruded high amylose maize starch. *Carbohydrate Polymers*, *80*(3), 699–710. https://doi.org/10.1016/j.carbpol.2009.12.001

Szymanska-Chargot, M., Chylinska, M., Gdula, K., Koziol, A., & Zdunek, A. (2017). Isolation and characterization of cellulose from different fruit and vegetable pomaces. *Polymers*, *9*(10). https://doi.org/10.3390/polym9100495

Van Soest, J. J. G., Hubertus, T., de Wit, D., & Vliegenthart, J. F. G. (1995). Short-range structure in (partially) crystalline potato starch determined with attenuated total reflectance Fourier-transform IR spectroscopy. *Carbohydrate Research*, *279*(C), 201–214. https://doi.org/10.1016/0008-6215(95)00270-7

Warren, F. J., Perston, B. B., Royall, P. G., Butterworth, P. J., & Ellis, P. R. (2013). Infrared spectroscopy with heated attenuated total internal reflectance enabling precise measurement of thermally induced transitions in complex biological polymers. *Analytical Chemistry*, *85*(8), 3999–4006. https://doi.org/10.1021/ac303552s
